# The FAIR database: facilitating access to public health research literature

**DOI:** 10.1093/jamiaopen/ooae139

**Published:** 2024-12-13

**Authors:** Zhixue Zhao, James Thomas, Gregory Kell, Claire Stansfield, Mark Clowes, Sergio Graziosi, Jeff Brunton, Iain James Marshall, Mark Stevenson

**Affiliations:** Department of Computer Science, University of Sheffield, Sheffield S10 2TN, United Kingdom; EPPI Centre, UCL Social Research Institute, Institute of Education, University College London, London WC1E 6BT, United Kingdom; School of Medicine and Population Health, University of Sheffield, Sheffield S10 2TN, United Kingdom; EPPI Centre, UCL Social Research Institute, Institute of Education, University College London, London WC1E 6BT, United Kingdom; Department of Population Health Sciences, School of Life Course & Population Sciences, Faculty of Life Sciences & Medicine, Kings College London, London WC2R 2LS, United Kingdom; EPPI Centre, UCL Social Research Institute, Institute of Education, University College London, London WC1E 6BT, United Kingdom; EPPI Centre, UCL Social Research Institute, Institute of Education, University College London, London WC1E 6BT, United Kingdom; School of Medicine and Population Health, University of Sheffield, Sheffield S10 2TN, United Kingdom; Department of Computer Science, University of Sheffield, Sheffield S10 2TN, United Kingdom

**Keywords:** evidence synthesis, research synthesis, public health, inequalities, machine learning, automatic database curation

## Abstract

**Objectives:**

In public health, access to research literature is critical to informing decision-making and to identify knowledge gaps. However, identifying relevant research is not a straightforward task since public health interventions are often complex, can have positive and negative impacts on health inequalities and are applied in diverse and rapidly evolving settings. We developed a “living” database of public health research literature to facilitate access to this information using Natural Language Processing tools.

**Materials and Methods:**

Classifiers were identified to identify the study design (eg, cohort study or clinical trial) and relationship to factors that may be relevant to inequalities using the PROGRESS-Plus classification scheme. Training data were obtained from existing MEDLINE labels and from a set of systematic reviews in which studies were annotated with PROGRESS-Plus categories.

**Results:**

Evaluation of the classifiers showed that the study type classifier achieved average precision and recall of 0.803 and 0.930, respectively. The PROGRESS-Plus classification proved more challenging with average precision and recall of 0.608 and 0.534. The FAIR database uses information provided by these classifiers to facilitate access to inequality-related public health literature.

**Discussion:**

Previous work on automation of evidence synthesis has focused on clinical areas rather than public health, despite the need being arguably greater.

**Conclusion:**

The development of the FAIR database demonstrates that it is possible to create a publicly accessible and regularly updated database of public health research literature focused on inequalities. The database is freely available from https://eppi.ioe.ac.uk/eppi-vis/Fair.

**NETSCC ID number:**

NIHR133603.

## Background and significance

Timely access to the research literature is vital for informing a wide range of public health decisions. However, important barriers exist. Public health interventions are often complex and applied in diverse and rapidly evolving settings. First, there is often a need to sift through many studies with diverse designs (typically thousands, but sometimes hundreds of thousands) to locate research that might be relevant to a specific decision.^1–3^ Second, health inequalities, which are defined as differences in health which are avoidable, remediable, and considered unjust,^4^ can be exacerbated by well-intentioned interventions.^5^ Thus, outcomes can be difficult to predict without a thorough understanding of the research literature. Understanding public health as a complex interconnected system increases the need to look holistically at the entirety of relevant evidence,^6^^,^^7^ though this also increases the volume of research to be examined.

Recent years have seen increasing research attention on the application of methods from Artificial Intelligence and Machine Learning to automate research synthesis, largely by applying them to the laborious parts of the evidence synthesis process such as identification of relevant studies,^8–10^ study appraisal,^11^ data extraction,^12^ and synthesis.^13^ To date, these methods have been largely focused on identifying and appraising randomized controlled trials, and usually in the context of speeding up systematic reviews of clinical healthcare interventions.^14^^,^^15^ However, there has been less focus on other areas of healthcare, such as public health, despite its importance and the significant challenges faced in access it.

## Objective

Our goal is to facilitate timely access to public health evidence with a focus on effects on health inequalities. Public health research is reported in a larger and more diverse body of scientific literature than has been evaluated in prior work on automation for evidence synthesis.

To achieve this objective, we develop methods to apply machine learning and Natural Language Processing approaches to support the review, assessment, evaluation, and summary of large volumes of public health research to support decision-making. We develop and apply automatic methods for identifying information about inequalities, study types, and common themes mentioned within this large body of research. These techniques are applied within a “living” online database that allows decision-makers to rapidly get to grips with making sense of a large volume of research on public health topics, including implications for health inequalities.

## Materials and methods

A stakeholder group was formed consisting of public health experts working within a range of settings including academia, local government, charities, and national bodies. The stakeholder group was consulted to ensure that the methods and tools developed met their needs.

Development of the database was broken down into multiple tasks:

1. Development and evaluation of supervised machine learning methods to identify health inequalities being discussed within research literature.
2. Development and evaluation of supervised machine learning methods to identify the study type being conducted.
3. Integration of these methods into a workflow to create a “living” database of public health research literature.

### Identification of health inequalities

PROGRESS-Plus is a conceptual framework that enables researchers to understand the social and personal factors which may influence health opportunities and outcomes.^16^ Many systematic reviews have applied an “equity lens” to their analysis by applying this tool to the studies included, and it has been recommended by the Campbell and Cochrane Equity Methods Group.^4^ The PROGRESS acronym refers to: Place of residence, Race/ethnicity/culture/language, Occupation, Gender/sex, Religion, Education, Socioeconomic status, and Social capital. Plus refers to: (1) personal characteristics associated with discrimination (eg, age, disability); (2) features of relationships (eg, smoking parents, excluded from school; and (3) time-dependent relationships (eg, leaving the hospital, respite care, other instances where a person may be temporarily at a disadvantage).

We use PROGRESS-Plus as the basic framework to classify research papers according to the inequality dimension being discussed. As a target task, we aim to predict which (if any) of the PROGRESS-Plus dimensions apply to a study. Note that we treat “Plus” as a single dimension, producing 9 categories (ie, the 8 PROGRESS-Plus categories and the Plus).

#### Assembly of data 

Automating the assignment of PROGRESS-Plus categories required annotated training data (ie, records that have already been categorized using the PROGRESS-Plus classification schema). We constructed this dataset by identifying systematic reviews in public health that present research described by the PROGRESS-Plus criteria and utilizing the data they contain from tables describing the characteristics of included studies. To find relevant reviews, we conducted searches of the following 14 resources during March 2021 for systematic reviews that used the PROGRESS-Plus tool: Campbell collaboration gap maps, Campbell Collaboration Journal, CINAHL Plus (EBSCO), Cochrane Library, Epistemonikos, Europe Pubmed Central, Google Scholar, MEDLINE (EBSCO), Microsoft Academic, NICE Evidence Search, Pubmed Central, Science Direct, SCOPUS, and Wiley online library. We also drew on work from unpublished EPPI-Centre research, consulted our advisory group, and advertised on social media.

Our searches retrieved 1360 records of research (after deduplication) to identify 177 reviews that used PROGRESS-Plus as part of their data extraction. We prioritized reviews that contained data in tables, where it was clear which studies corresponded to the PROGRESS-Plus categories and the studies had a bibliographic citation. For some reviews, the full-text report or supplementary data did not contain tables that could be reused (ie, provided bibliographic information about studies along with the PROGRESS-Plus categories they employed). The authors of 12 such reviews containing over 50 studies were contacted to ask if their data could be supplied in a format that was straightforward to reuse. This approach allowed us to leverage the large amount of existing data from 66 reviews, rather than labeling new texts from scratch.

Two professional public health researchers were each randomly assigned a subset of the 66 reviews and used the information within them to identify the PROGRESS-Plus category that applied to each study, where this was clearly stated within the review. No interpretative work was involved in this task, which simply consisted of the copying and pasting of tabular data out of the paper and into Excel. This information was then used to provide labels for each study for machine learning. A single study can be labeled with multiple PROGRESS-Plus categories, so that a single study can have anywhere between zero and 9 labels applied to it. In total, 1978 studies were labeled this way. A significant variance was observed in the frequency with which PROGRESS-Plus categories were assigned to studies. Categories were assigned as follows (in descending order of frequency): Gender (48.28%), Place (42.06%), Race (41.25%), Socioeconomic (39.08%), Plus (29.68%), Education (24.57%), Occupation (14.51%), Social capital (6.77%), and Religion (2.83%). (The dataset is available from https://eppi.ioe.ac.uk/cms/Default.aspx?tabid=3830.)

#### Computational methods

Classifiers were developed based on BERT with a linear classification layer using the HuggingFace platform.^17^ BERT was chosen to support the long-term stability of the database since it allows a classifier to be developed and run locally with the rest of the database. Large language models, such as Gemini and GPT-4, have recently demonstrated strong performance on similar tasks. However, their performance has increased substantially since the work reported here was conducted, and at the time, they were not commonly considered as suitable for this task. (Please also see the Discussion section for more information on large language models.)

The available data were split into a training and evaluation dataset in a 4:1 ratio. Two approaches were compared, one treated the task as a multilabel classification problem where each data point can be associated with none, one or multiple categories (multilabel_bert), and the second which used 9 separate binary classifiers to identify each category (binary_bert). The input in both evaluations was a concatenation of the titles and abstracts of the training data, and we also experimented with including the journal title. The maximum length of input data BERT can currently process is 512 tokens. The overwhelming majority of abstracts were under this limit; for longer abstracts, we truncated to the first 512 tokens.

### Identification of research design

Evidence of potential interest to public health decision-makers includes a diverse range of study types. An existing tool^14^ was extended to classify each research record according to the type of study being described with 8 types being used: Cohort study, Consensus study, Clinical trial, Protocol, Guideline, Qualitative study, Randomized controlled trial (RCT), and Systematic review.

The data used to predict the above types of study were assembled from MEDLINE (accessed via the PubMed), with title/abstract records indexed using selected study design tags from the MeSH and Publication Type fields. These indexes have, primarily, been manually applied to articles added to MEDLINE by staff at the US National Library of Medicine (NLM). From 2016, an automated tool has been used on a limited scale to index some new records; and the NLM proposes to automatically index all records by 2026. The dataset was limited to manually indexed articles.

Given the raw PubMed dataset is large (>30 million records) and there is an overwhelming class imbalance with relevant articles being a tiny minority (eg, trial protocols represent <0.01% of all articles), a pragmatic strategy was applied to create workable datasets for machine learning use. All abstracts which were indexed with the following study designs were included as positive examples (figure in brackets indicates number of examples): Randomised Control Trials (489 295); Systematic Reviews (121 063); Case-Control Studies (65 790); Clinical Trials (593 277); Cohort Studies (78 688); Guidelines (33 826); Consensus Development Conferences (10 093); Qualitative Research (7577); and Clinical Trial Protocols (3235). Negative examples were obtained by randomly sampling from articles that did not have the relevant index to create a 1:1 ratio of positive and negative articles in each dataset.

An 80/10/10 split was created through random sampling for the training, validation, and test datasets. The hyperparameters of all the models, bar the clinical trial classifier, were optimized via a cross-validated grid-search on the training data, while performance was evaluated on the test data. The validation set was created beforehand for optimizing the hyperparameters of future models and to ensure consistency in the data used between experiments.

To develop and evaluate the study design classification, the data were again split randomly in a 4:1 ratio for training and evaluation, respectively. A binary “bag-of-words” method was used to convert text to numeric vectors, with “stop-words” (very common words with low informational content, such as “and,” “but,” or “of”) removed as a preprocessing step. Our bag-of-words representation considered *n-grams* (consecutive word sequences) ranging from 1 to 4 in length. We used a series of logistic regression models with L2 regularization to model each study design under consideration. We implemented the vectorization using a hashing vectorizer, and models using stochastic gradient descent; both using the Scikit-learn package in Python.

### Development of online database

**Figure 1.**
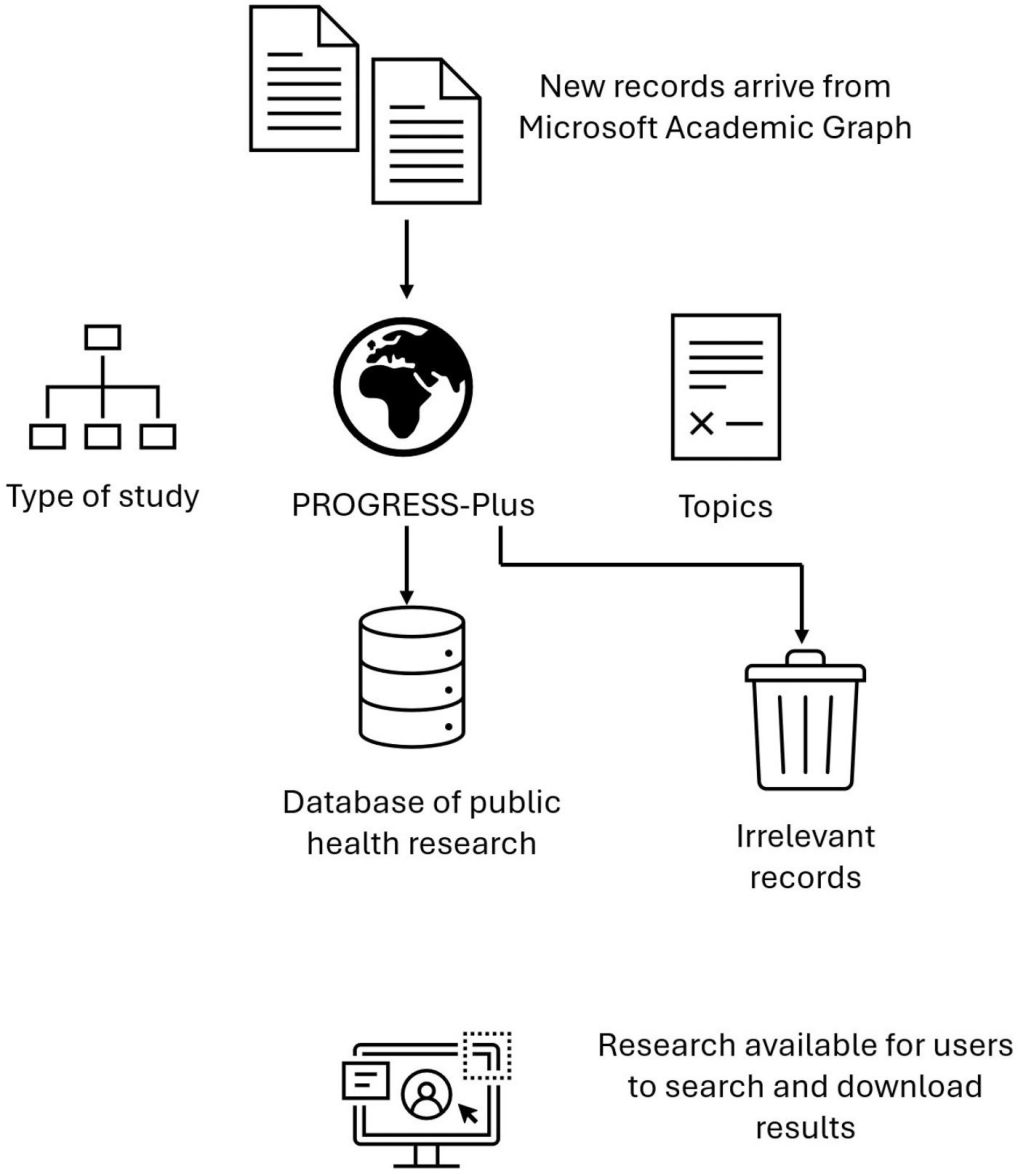
FAIR database workflow.

The 2 models described above were used within the development of an online tool, the FAIR (**F**inding **A**ccessible **I**nequalities **R**esearch) Database. (This name was selected because it combines the ethical imperative of health equity with the FAIR acronym which is increasingly used to describe good practice in sharing research data (being Findable, Accessible, Interoperable, and Reusable): https://www.go-fair.org/fair-principles/.) The overall workflow of the database is summarized in Figure 1.

#### Identification of relevant studies

Records for inclusion in the database are first identified using a combination of Microsoft Academic “Field of Study” (topic) searches (using “health equity” and “social determinants of health”) and machine learning tools which have been previously described for locating references when maintaining a surveillance of COVID-19 (coronavirus disease 2019) research.^18^

#### Automatic classification

Open Alex does not provide information about study type or PROGRESS-Plus categories so this information is added to relevant records using the tools described earlier. In addition, included studies were also associated with selected categories from the Wikipedia hierarchy which provides additional information beyond the study type and PROGRESS-Plus categories. Wikipedia was chosen since we found that topics in public health, particularly those concerned with health inequalities, to be well developed and well structured. The use of Wikipedia allows the included studies to be navigated using an existing hierarchical structure that may be familiar to the database’s intended users. For example, to identify studies in the database related to a particular disease of interest. An additional benefit is that Wikipedia provides summary text and (in many cases) illustrative images for concepts in the hierarchy which can support navigation. The Wikipedia hierarchy was analyzed and a set of candidate Wikipedia categories identified. It was observed that those “public health,” “social inequality,” “diseases of poverty,” “neglected tropical diseases,” and “tropical disease” in the hierarchy provided good coverage of the database’s subject matter, although they did not cover common diseases. These were added using a list available from the NHS Scotland Inform website (https://www.nhsinform.scot/illnesses-and-conditions/a-to-z). Studies were matched onto this set of categories using a simple matching method based on the similarity of the text of the study and Wikipedia page. We were unable to directly evaluate this approach due to a lack of gold-standard data.

The FAIR database is implemented within EPPI-Reviewer, existing software infrastructure developed at UCL’s EPPI-Centre. It is a “review” (or “map”) within this software and configured so that new records are automatically added every 2 weeks then analyzed using the automatic classification techniques which are implemented within Microsoft’s Azure architecture.^19^ The database was populated with records identified from the >240 million records in the Microsoft Academic dataset and now maintained with a feed of records from the OpenAlex dataset. (https://openalex.org/. The OpenAlex dataset was designed to replace Microsoft Academic which closed at the end of 2021.)

## Results

### Health inequality identification

Accuracy of Progress-Plus identification was evaluated using standard metrics: recall, precision, F1, and area under the receiver operating characteristics curve (AUROC). Four models were created: binary_bert and multilabel_bert which treat the problem as a binary/multilabel classification task respectively (see above) and binary_bert (with journal) and multilabel_bert (with journal) which make use of the journal title in addition to the title and abstract.

**Figure 2.**
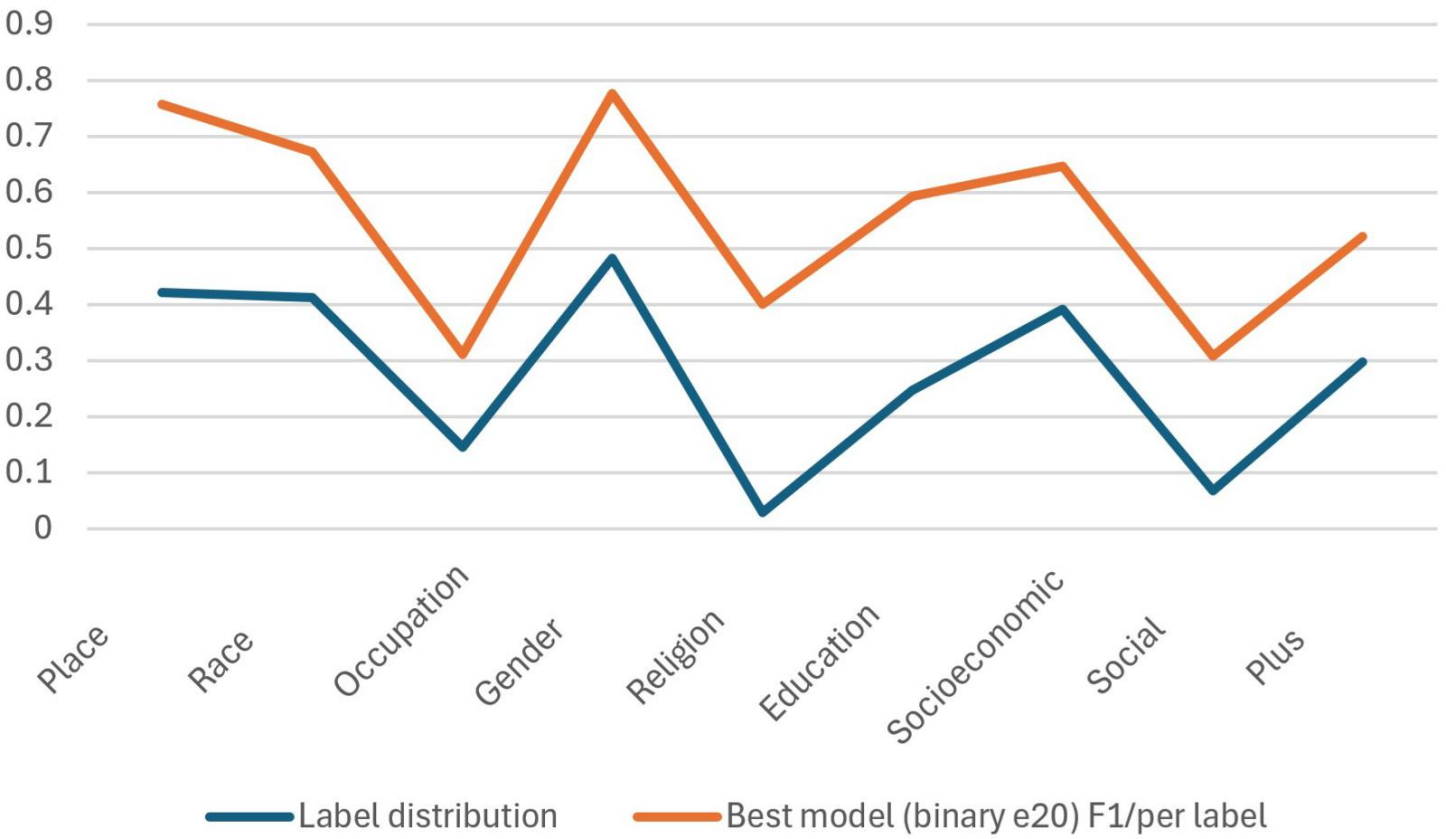
Association between number of labeled examples available and model performance across PROGRESS-Plus categories.

Performance of these 4 models is shown in Table 1. All approaches achieved micro averaged F1 scores in the range 0.62-0.65. However, average conceals considerable variation in performance between PROGRESS-Plus categories. Table 2 shows that performance for all metrics varied considerably between categories, with the best F1 scores (in bold) varying between 0.308 (for the Social category) and 0.777 (for Gender). This difference in performance can largely be attributed to the variation in the distribution of PROGRESS-Plus categories in the dataset, which affects the number of examples available to train the model (see Figure 2).

**Table 1.** PROGRESS-Plus classification performance.

Model	micro F1	macro F1	macro AUROC
binary_bert	0.647 (0.646, 0.648)	**0.544 (0.542, 0.546)**	**0.693** (0.692, 0.694)
binary_bert (with journal)	0.620 (0.619, 0.621)	0.505 (0.503, 0.507)	0.674 (0.673, 0.675)
mutlilabel_bert	**0.651 (0.650, 0.652)**	0.481 (0.479, 0.482)	0.670 (0.670, 0.671)
mutlilabel_bert (with journal)	0.649 (0.648, 0.650)	0.521 (0.519, 0.523)	0.681 (0.680, 0.682)

**Table 2.** Per-category performance of PROGRESS-Plus classification.

	Label count	Label proportion	Recall	Precision	Best model F1
Place	844	0.421	0.756 (0.754, 0.758)	**0.760 (0.758, 0.762)**	0.758 (0.756, 0.760)
Race	831	0.413	0.680 (0.678, 0.682)	0.667 (0.665, 0.669)	0.673 (0.671, 0.675)
Occupation	300	0.145	0.241 (0.237, 0.245)	0.438 (0.433, 0.443)	0.311 (0.307, 0.315)
Gender	983	0.483	**0.793 (0.791, 0.795)**	0.760 (0.758, 0.762)	**0.777 (0.776, 0.778)**
Religion	59	0.028	0.429 (0.419, 0.439)	0.375 (0.355, 0.395)	0.400 (0.387, 0.413)
Education	513	0.246	0.594 (0.591, 0.597)	0.594 (0.591, 0.597)	0.594 (0.591, 0.597)
Socioeconomic	794	0.391	0.690 (0.688, 0.692)	0.609 (0.607, 0.611)	0.647 (0.645, 0.647)
Social capital	155	0.068	0.211 (0.206, 0.216)	0.571 (0.560, 0.582)	0.308 (0.302, 0.314)
Plus	619	0.297	0.415 (0.412, 0.418)	0.700 (0.697, 0.703)	0.521 (0.519, 0.523)

### Study design classification

The other supervised machine learning models in this task were those that denoted the study design of research in the database. Again, we evaluated F1, recall, precision, and AUROC.

Table 3 shows the performance of the study type design models. Overall, they are consistently accurate with the statistics for the systematic review and protocol models being particularly good. At the other end of the scale, the model has had more difficulty with Guidelines; while recall is good, precision is notably lower for this type of study.

**Table 3.** Performance of study design classifier models.

	Precision	Recall	F1	AUCROC
RCT	0.889 (0.887-0.892)	0.950 (0.948-0.952)	0.919 (0.916-0.921)	0.923
Systematic review	0.965 (0.962-0.968)	0.986 (0.983-0.988)	0.975 (0.973-0.978)	0.99
Case control study	0.808 (0.799-0.817)	0.889 (0.881-0.897)	0.847 (0.838-0.855)	0.92
Clinical trial	0.768 (0.765-0.771)	0.855 (0.852-0.857)	0.809 (0.806-0.812)	0.821
Cohort study	0.813 (0.804-0.821)	0.863 (0.855-0.870)	0.837 (0.829-0.845)	0.844
Guideline	0.457 (0.446-0.469)	0.955 (0.948-0.962)	0.618 (0.606-0.631)	0.847
Consensus study	0.653 (0.629-0.678)	0.933 (0.917-0.948)	0.768 (0.746-0.791)	0.838
Qualitative study	0.888 (0.867-0.910)	0.951 (0.934-0.965)	0.918 (0.899-0.937)	0.919
Protocol	**0.982 (0.971-0.998)**	**0.991 (0.980-1.000)**	**0.986 (0.976-0.999)**	0.986

### FAIR database

**Figure 3.**
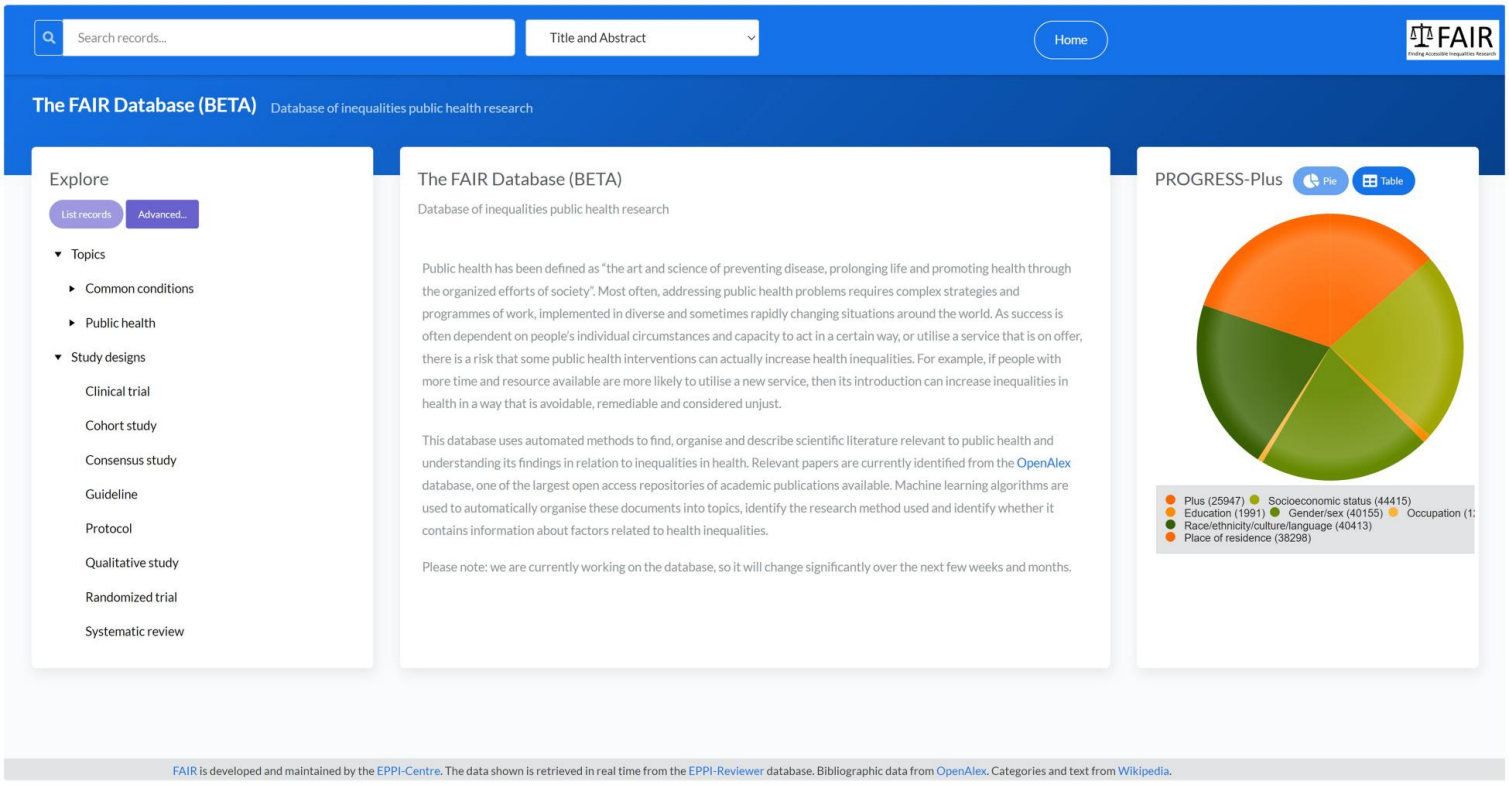
FAIR home page.

**Figure 4.**
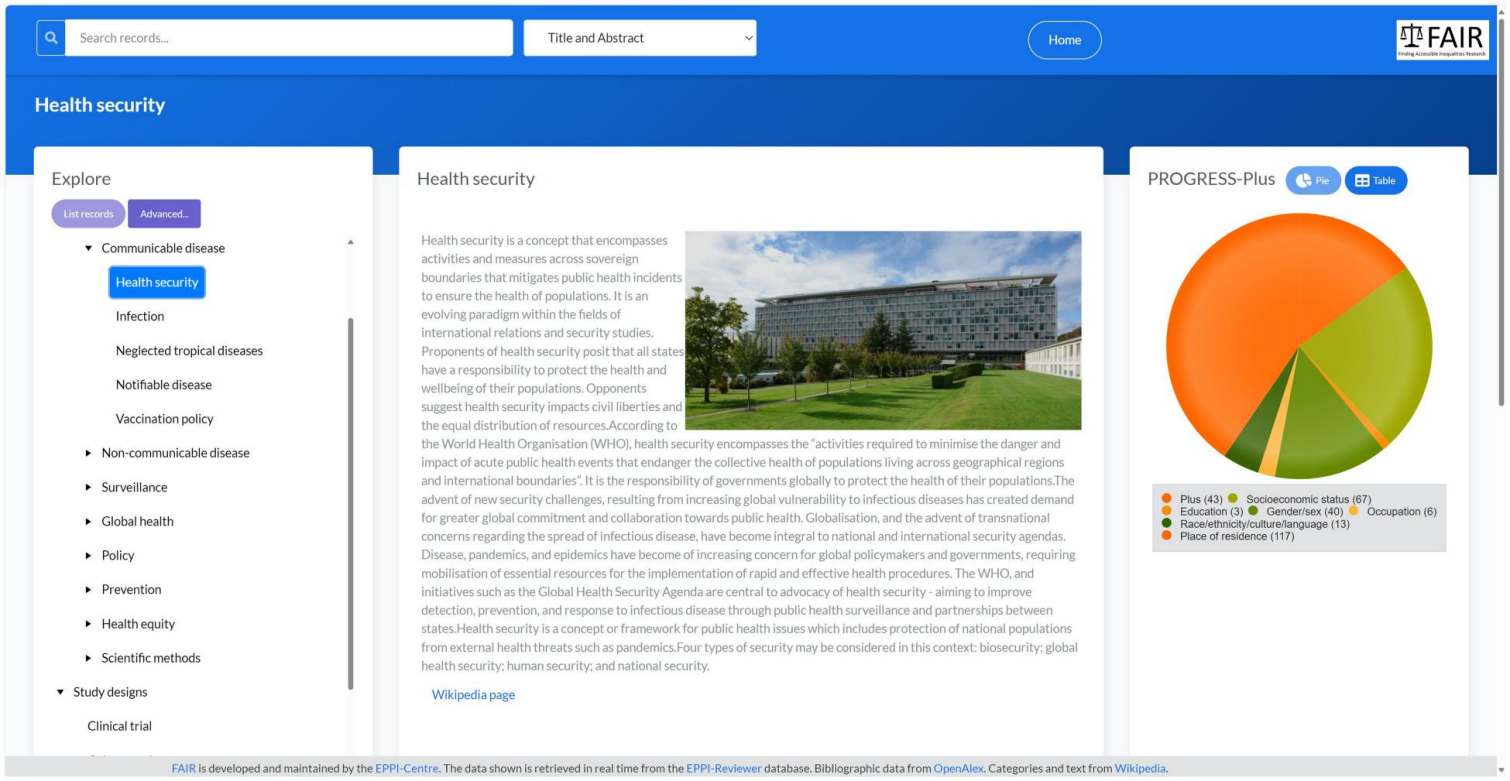
A FAIR “topic” page.

The homepage for the FAIR database is shown in Figure 3. On the right is a pie chart showing the distribution of research according to the PROGRESS-Plus criteria. Clicking any segment of the pie chart will result in the records in the selected category being listed. On the left are the Wikipedia categories organized into a browsable hierarchy along with the various study designs modeled in Task 1. If a user selects a category on the left and clicks “list records,” then a “topic” page appears, containing information from Wikipedia, and the records assigned to that category (Figure 4). “Sensemaking” is supported by the summary information from the Wikipedia page along with a graphic to break up the text. The pie chart summarizing PROGRESS-Plus information is now filtered to include only research within the selected topic. Users can interact with the research on the topic using the list at the bottom of the screen.

The categories on the left can be combined using Boolean AND NOT to query the database in flexible ways, and free text searches on titles, abstracts, authors, and publication year can be conducted using the text entry box at the top of the screen.

**Figure 5.**
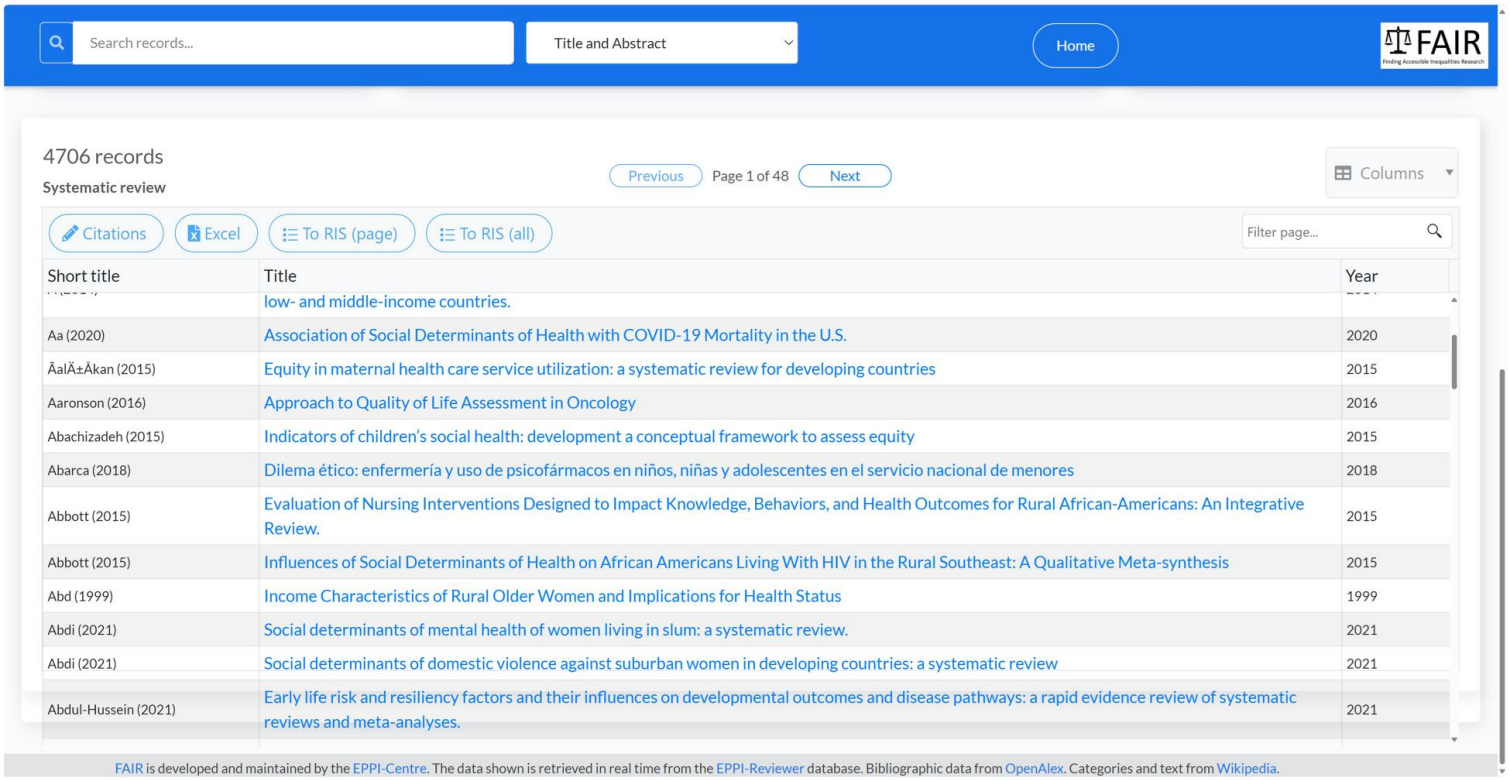
List of search results.

All lists of results take a standard form, no matter which method was used to identify them, allowing users to download the records in text, spreadsheet, and RIS form (Figure 5).

**Figure 6.**
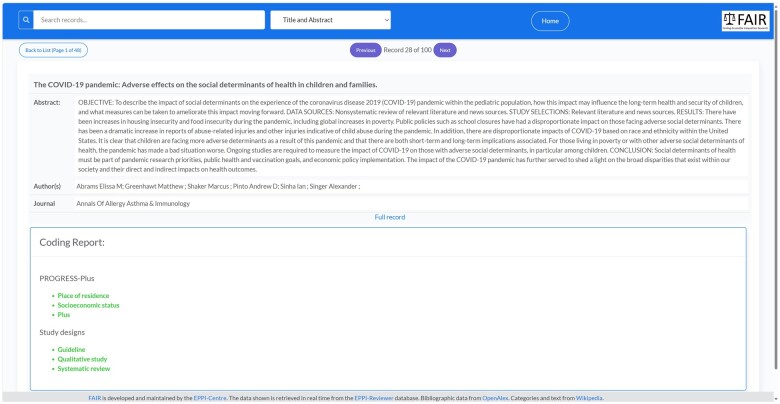
Example of codes applied to an individual record.

Finally, users can examine any individual record in detail. This screen provides full bibliographical information and links for further information, including the automatically applied classifications of study type, PROGRESS-Plus category, study type, and Wikipedia category (Figure 6).

The database currently (February 2024) contains over 192 000 records and is continually updated as new studies are published.

## Discussion

Development of FAIR demonstrates that it is possible to develop and apply NLP (Natural Language Processing)-based automation technologies to create a publicly accessible and regularly update database focused on inequalities in health. The research it contains augments other processes of finding information and is accessible in resource-limited contexts. This work represents a much needed corrective to the way that automation techniques are currently focused on medical interventions.^20^^,^^21^ The challenges in public health are arguably greater, but this area has received much less attention, increasing the relative costs of doing evidence synthesis of public health research in comparison to more clinical areas. Other tools to support discovery of research, such as scholarly bibliographic databases may lie behind expensive paywalls, may not contain the breadth of research available from OpenAlex, and are sometimes not well-indexed and difficult to use.

The creation of this database has been supported by the development of tools to identify and classify public health research related to inequalities, particularly study type and PROGRESS-Plus category. The study design classifier proved to be accurate in most cases which was expected since this problem has already been explored and a large volume of training data is readily available from PubMed. However, identifying PROGRESS-Plus categories proved to be more challenging. For example, the “Social capital” and “Religion” categories have not been applied to any studies. There are multiple potential causes for the lower accuracy of the PROGRESS-Plus classifier: (1) the relatively small volume of labeled training data available; (2) the primary research studies used to derive this annotated data were developed by different teams working on substantive reviews, so it is quite possible that the PROGRESS-Plus criteria have been applied differently in different reviews; and (3) information relevant to the assignment of PROGRESS-Plus categories may not always be present in the titles and abstracts of records. The annotated dataset created to support the development of the PROGRESS-Plus classifier will support future exploration of these possible causes. However, even noisy class labels, such as those provided by the PROGRESS-Plus classifier, may prove to be useful within an exploratory search environment such as the FAIR database since they offer the user some information about the domain. In addition, it would be useful for a future study to explore the performance of large language models for this task. While it seems unlikely that they would be able to overcome the main barrier to classification that we identified—the lack of detail in the abstracts—they may be able to bring some improvement in performance (though possibly marginal).

## Conclusions

This article described the development of the FAIR database which is designed to facilitate access to the diverse evidence base in the field of public health. This tool enables researchers, policymakers, and practitioners to identify relevant research to inform decision-making in this area. The database is freely available and regularly updated. It demonstrates that automation tools for evidence analysis can be developed and applied to public health research, as well as the clinical domains they are more commonly used in. We hope that the database is a useful resource for those who need to access the public health research literature.

We plan to continue to develop the database and to refine the automation tools underpinning it. In particular, we plan to explore methods for improving the assignment of PROGRESS-Plus categories and used feedback from users to inform future development.

## Data Availability

All data are freely available in the FAIR database. We would like to release all code as open source, and are currently seeking permission from NIHR to do so. Links will be found here: https://eppi.ioe.ac.uk/fair/.

## Open access

For the purpose of open access, the authors have applied a Creative Commons Attribution (CC BY) license to any Author Accepted Manuscript version arising from this submission.
